# Retrospective evaluation of Selexipag monotherapy on pulmonary hemodynamics in newly diagnosed, treatment-naïve patients with chronic thromboembolic pulmonary hypertension prior to balloon pulmonary angioplasty

**DOI:** 10.1016/j.ijcha.2026.101956

**Published:** 2026-06-15

**Authors:** Naohiro Komura, Teruyasu Sugano, Toru Suzuki, Fumiaki Ono, Maria Abe, Uno Yamamoto, Keisuke Sekiya, Sho Kodama, Shintaro Minegishi, Shingo Kato, Kozo Okada, Junya Hosoda, Masaaki Konishi, Fumiyuki Otsuka, Noriaki Iwahashi, Tomoaki Ishigami, Daisuke Utsunomiya, Makoto Mo, Kiyoshi Hibi

**Affiliations:** a Department of Cardiology, Yokohama City University Hospital; 3-9 Fukuura, Kanazawa-ku, Yokohama 236-0004, Japan; b Department of Cardiovascular Surgery, Yokohama Minami Kyosai Hospital; 1-21-1 Mutsuurahigashi, Kanazawa-ku, Yokohama 236-0037, Japan; c Department of Diagnostic Radiology, Yokohama City University Hospital; 3-9 Fukuura, Kanazawa-ku, Yokohama 236-0004, Japan; d Division of Cardiology, Yokohama City University Medical Center; 4-57 Urafune-cho, Minami-ku, Yokohama 232-0024, Japan

**Keywords:** Selexipag, Chronic thromboembolic pulmonary hypertension, Balloon pulmonary angioplasty, Riociguat

## Abstract

**Background:**

Selexipag has been approved for the treatment of chronic thromboembolic pulmonary hypertension (CTEPH) in Japan since April 2021; however, its hemodynamic effects in treatment-naïve patients with inoperable CTEPH prior to balloon pulmonary angioplasty (BPA) remain unclear. This study evaluated the impact of selexipag monotherapy on pulmonary hemodynamics before first BPA.

**Methods:**

Between August 2021 and February 2024, 40 newly diagnosed, treatment-naïve patients with inoperable CTEPH undergoing their initial BPA session were screened at Yokohama City University Hospital. After excluding three patients in the riociguat group due to insufficient titration time, 30 patients (15 selexipag, 15 riociguat) were included in this retrospective observational study. Hemodynamic parameters were assessed before and after pharmacotherapy and compared within and between groups.

**Results:**

The selexipag group included 10 women (mean age 66.5 years; average dose 2.2 mg/day). Selexipag significantly reduced mean pulmonary arterial pressure (mPAP) from 39.0 to 33.6 mmHg (−5.4 ± 6.7 mmHg; *P* = 0.008) and pulmonary vascular resistance (PVR) from 8.07 to 6.69 Wood units (WU; −1.38 ± 1.2 WU; *P* = 0.001), without a significant change in cardiac output (CO). Riociguat significantly reduced mPAP (−7.8 ± 8.5 mmHg; *P* = 0.003) and PVR (−2.57 ± 2.4 WU; P = 0.001) and increased CO (+0.81 ± 1.0 L/min; *P* = 0.006). Changes in mPAP and PVR did not differ significantly between groups, whereas CO increased significantly more with riociguat (*P* = 0.04).

**Conclusions:**

In this exploratory, hypothesis-generating retrospective study, selexipag monotherapy was associated with reductions in mPAP and PVR in treatment-naïve patients with inoperable CTEPH before BPA, whereas riociguat showed greater CO improvement. These findings warrant prospective validation in selected patients before broader clinical application.

## Graphical abstract

**Figure:**
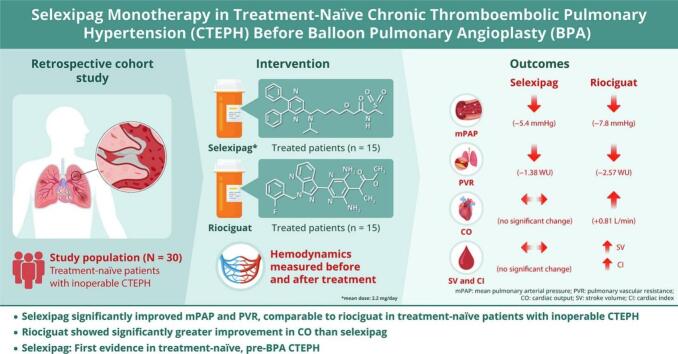

## 1 Introduction

Chronic thromboembolic pulmonary hypertension (CTEPH) is classified as Group-4 pulmonary hypertension (PH), characterized by persistent thromboembolic obstruction and secondary small-vessel vasculopathy, which progressively elevates pulmonary vascular resistance (PVR) and leads to severe PH, right heart failure, and death if left untreated [1], [2], [3].

Pulmonary endarterectomy (PEA) is the gold-standard and potentially curative treatment for CTEPH [4], [5]. However, approximately 40% of patients are ineligible for PEA due to distal lesions or significant comorbidities [6].

The management of inoperable CTEPH has evolved over the past decade, incorporating both PH-targeted medical therapy and balloon pulmonary angioplasty (BPA), a minimally invasive endovascular intervention [7], [8], [9]. BPA has emerged as a promising option for patients with inoperable CTEPH [8], [9], [10]. However, elevated baseline mean pulmonary arterial pressure (mPAP) and PVR are associated with increased risks of BPA-related lung injury, hemoptysis, and hemodynamic instability [11], [12], [13]. Therefore, pre-procedural hemodynamic optimization is considered clinically important to enhance BPA safety and outcomes. The 2022 ESC/ERS PH Guidelines endorse the use of PH-targeted medical therapy before BPA in suitable inoperable candidates (Class IIa, Level B) [14]. Supporting this concept, the RACE randomized trial demonstrated that riociguat pretreatment followed by BPA was associated with numerically fewer BPA-related complications than initial BPA alone [11].

In Japan, two PH-targeted drugs—riociguat and selexipag—are currently available for inoperable CTEPH. Riociguat, the first approved medical therapy for inoperable CTEPH, demonstrated improvements in exercise capacity, hemodynamics, and World Health Organization functional class (WHO-FC) in a pivotal clinical study [15]. Furthermore, two randomized controlled trials, RACE and MR-BPA, supported the efficacy of riociguat in treatment-naïve patients with inoperable CTEPH [10], [11].

Selexipag, an oral selective prostacyclin receptor agonist, was shown to reduce morbidity and mortality events in patients with pulmonary arterial hypertension (PAH) in the GRIPHON study [16]. In the context of CTEPH, a placebo-controlled phase 2 study in Japanese patients demonstrated a significant reduction in PVR with selexipag compared with placebo [17]. Furthermore, a phase 3, multicenter, randomized, placebo-controlled trial confirmed hemodynamic improvements and favorable tolerability in Japanese patients with inoperable CTEPH or persistent/recurrent PH following PEA and/or BPA [18]. Based on these findings, selexipag has been approved in Japan since April 2021 and subsequently utilized in clinical practice.

However, these prior selexipag studies enrolled heterogeneous populations, including patients receiving other PH-targeted drugs or with a history of BPA and/or PEA. Consequently, the hemodynamic effects of selexipag monotherapy in newly diagnosed patients prior to BPA remain unclear. To address this gap, we conducted a retrospective study to evaluate the hemodynamic impact of selexipag monotherapy in newly diagnosed, treatment-naïve, inoperable CTEPH patients prior to BPA, and to compare these hemodynamic outcomes with those of patients treated with riociguat.

## 2 Methods

### 2.1 Study cohort

**Fig. 1:**
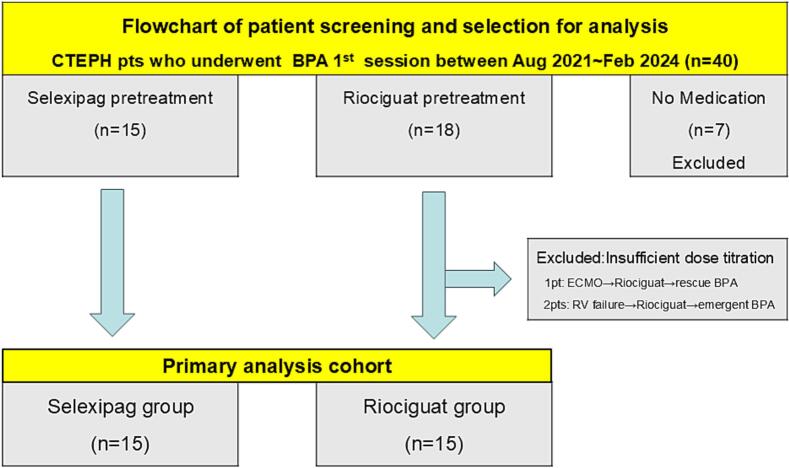
**Flowchart of the study.** Between August 2021 and February 2024, 40 newly diagnosed, treatment-naïve patients with inoperable CTEPH were screened. Seven patients who did not receive PH-targeted medication were excluded. Of the remaining 33 patients, 15 received selexipag and 18 received riociguat. Three riociguat-treated patients were excluded because BPA was performed before adequate dose titration, resulting in 15 patients per group for the final analysis.

This was a single-center, retrospective observational study conducted at Yokohama City University Hospital. A total of 40 consecutive patients with newly diagnosed, treatment-naïve inoperable CTEPH who were scheduled to undergo their first BPA session between August 2021 and February 2024 were screened. Among them, 15 patients received selexipag pretreatment and 18 received riociguat pretreatment. The remaining 7 patients proceeded directly to BPA without PH-targeted pretreatment and were excluded. In the riociguat group, 3 patients required urgent BPA due to clinical deterioration (1 requiring extracorporeal membrane oxygenation [ECMO]-supported rescue BPA and 2 undergoing emergent BPA for severe right ventricular failure) and were excluded because dose titration was insufficient. Therefore, the final study cohort consisted of 30 patients who received monotherapy with either selexipag (*n* = 15) or riociguat (n = 15) prior to the first BPA session. The patient screening and selection process is illustrated in Fig. 1.

Operability for CTEPH was evaluated by our multidisciplinary CTEPH team, which consisted of an experienced PEA surgeon (MM), BPA interventionists (NK, TS), PH specialists (FO, TSz), and thoracic radiologists (DU, SKa). “Inoperable CTEPH” was defined as CTEPH in patients who met at least one of the following criteria: (1) technically inaccessible thromboembolic lesions, (2) significant comorbidities and/or frailty precluding PEA, or (3) patient refusal after multidisciplinary evaluation. In our cohort, 20 patients met criterion (1), 10 met criterion (2), and none met criterion (3).

Drug selection was determined by our dedicated multidisciplinary CTEPH team, considering drug–drug interactions, comorbidities, systemic blood pressure, and overall hemodynamic status. During the study period, combination PH-targeted therapy prior to BPA was not routinely implemented at our institution; therefore, all patients received monotherapy before BPA. This clinical practice allowed a direct comparison of the pharmacological effects of selexipag and riociguat without the confounding influence of combination therapy.

Right heart catheterization (RHC) was performed at the time of CTEPH diagnosis and immediately before the first BPA session in both groups. Pulmonary hemodynamics obtained from these two assessments were used to evaluate the treatment effects of selexipag and riociguat.

This study was conducted in accordance with the ethical principles of the Declaration of Helsinki and was approved by the institutional ethics committee of Yokohama City University Hospital (approval number F230500041, June 6, 2023). The requirement for written informed consent was waived owing to the retrospective study design, and information regarding the study was disclosed to all patients via an opt-out approach.

### 2.2 CTEPH diagnosis and treatment protocol at our hospital

In accordance with the Japanese guidelines used during the study period [19], CTEPH was diagnosed based on characteristic findings from at least two imaging modalities (ventilation–perfusion scintigraphy, computed tomography pulmonary angiography, and/or catheter-based pulmonary angiography) together with hemodynamic confirmation by RHC (mPAP ≥25 mmHg and pulmonary arterial wedge pressure [PAWP] ≤15 mmHg at rest).

Previous studies have demonstrated that patients with mPAP ≥30 mmHg have a poorer prognosis [20], [21] and a higher risk of BPA-related complications [12], [13]. Based on these findings, our multidisciplinary CTEPH team considered mPAP ≥30 mmHg as a high-risk condition and initiated PH-targeted monotherapy with selexipag or riociguat prior to BPA whenever clinically appropriate.

Dosing and titration of riociguat followed the approved Japanese package insert, which is consistent with the dosing regimen used in the CHEST-1 trial (initiated at 1.0 mg three times daily [TID] and up-titrated by 0.5 mg every 2 weeks up to 2.5 mg TID, as tolerated) [15]. Selexipag was initiated at 0.2 mg twice daily (BID) and up-titrated by 0.2 mg increments at intervals of ≥7 days up to 1.6 mg BID, in accordance with the Japanese package insert for CTEPH (Uptravi®), which adopts the same titration scheme as the global PAH label and as used in the GRIPHON trial [16]. Dose escalation for both agents was withheld in the presence of adverse effects such as hypotension.

All patients with CTEPH received lifelong anticoagulant therapy with either warfarin or a direct oral anticoagulant, in accordance with current clinical practice. Patients were hospitalized for three nights and four days for each BPA session, and RHC was performed at the beginning of the first session.

For hemodynamic assessment, RHC was performed using a Swan–Ganz catheter (Edwards Lifesciences®). Hemodynamic measurements were obtained in the supine position without supplemental oxygen after at least 10 min of rest and recorded at end-expiration to ensure consistency in pre- and post-treatment evaluations. Cardiac output (CO) was measured using the thermodilution method.

### 2.3 Study outcomes

We conducted a comparative analysis of pulmonary hemodynamic and oxygenation parameters before and after PH-targeted treatment, including mPAP, right atrial pressure (RAP), PAWP, CO, stroke volume (SV), heart rate (HR), cardiac index (CI), mixed venous oxygen saturation (SvO₂), arterial oxygen saturation (SaO₂), and PVR. All parameters were measured during RHC. In addition, we compared the changes in these parameters between the selexipag and riociguat groups. Adverse effects commonly associated with both drugs were also reviewed.

### 2.4 Statistical analysis

Continuous variables were expressed as mean ± standard deviation (SD) or median (interquartile range [IQR]), as appropriate. Baseline hemodynamic parameters, including mPAP, PVR, and CO, were compared between groups using independent Student's *t*-tests or the Mann–Whitney *U* test, as appropriate. Categorical variables were compared using the chi-square test or Fisher's exact test, as appropriate. Within-group hemodynamic changes before and after treatment were assessed using paired Student's *t*-tests, and between-group differences in Δ-values were evaluated using independent Student's t-tests.

To ensure valid between-group comparisons beyond Δ-value t-tests, analysis of covariance (ANCOVA) was performed for each hemodynamic parameter, adjusting for the corresponding baseline value, age, sex, WHO-FC, and the interval between baseline and pre-BPA RHC. Model assumptions were evaluated by visual inspection and the Shapiro–Wilk test to confirm residual normality. Adjusted mean changes and between-group *P*-values were reported.

For variables (including Δ-values) that violated normality assumptions, non-parametric tests (Wilcoxon signed-rank test or Mann–Whitney *U* test) were performed as sensitivity analyses to confirm the robustness of the findings. A two-tailed P-value <0.05 was considered statistically significant. All statistical analyses were performed using SPSS version 22 software (IBM Corp., Armonk, NY, USA).

## 3 Results

### 3.1 Baseline characteristics and safety outcomes

A total of 30 patients were included in the comparative analysis, comprising 15 in the selexipag group and 15 in the riociguat group (Fig. 1). Baseline demographic and clinical characteristics, including age, sex, WHO-FC, B-type natriuretic peptide, 6-min walk distance, and hemodynamic parameters, were comparable between the two groups (Table 1). The median interval between baseline and pre-BPA hemodynamic assessments was also similar between the groups (136 [113–249] vs. 112 [99–179] days).

**Table 1:** Demographic data and comorbidities of patients.

	**Selexipag** (*n* = 15)	**Riociguat** (n = 15)	**P-value**
Age, years	66.5 ± 10.6	72.0 ± 13.7	0.23
Sex, male, n (%)	5 (33)	7 (47)	0.46
**Clinical severity**			
WHO-FC II / III, n (%)	8 (53) / 7 (47)	10 (67) / 5 (33)	0.71
BNP, pg/mL (median, IQR)	74 (32–183)	109 (24–242)	0.98
6MWD, m	360 ± 124	386 ± 114	0.58
Interval between assessments, days (median, IQR)	136 (113–249)	112 (99–179)	0.21
**Anticoagulation**			
Direct oral anticoagulant, n (%)	13 (87)	13 (87)	1.00
Warfarin, n (%)	2 (13)	2 (13)	1.00
**Comorbidities**			
Acute pulmonary embolism	9 (60)	11 (73)	0.44
Dyslipidemia	6 (40)	3 (20)	0.23
Hypertension	7 (47)	8 (53)	0.72
Mental illness	3 (20)	2 (13)	0.62
Malignant tumor	3 (20)	4 (27)	0.67
Diabetes	3 (20)	0 (0)	0.07
Antiphospholipid antibody syndrome	1 (7)	1 (7)	1.00
Coronary artery disease	1 (7)	0 (0)	0.31
**Hemodynamics at baseline**			
mPAP, mmHg	39.0 ± 7.7	40.8 ± 9.3	0.57
RAP, mmHg	6.1 ± 2.0	6.3 ± 3.7	0.90
PAWP, mmHg	8.4 ± 2.8	8.7 ± 3.4	0.82
CO, L/min	4.19 ± 1.2	4.03 ± 0.8	0.66
CI, L/min/m^2^	2.50 ± 0.6	2.47 ± 0.4	0.90
PVR, WU	8.07 ± 3.4	8.18 ± 3.0	0.93

Data are presented as mean ± standard deviation or median (interquartile range), as appropriate. Categorical variables are shown as numbers and percentages. Comparisons were performed using Student's t-test or Mann–Whitney U test for continuous variables and Fisher's exact test for categorical variables. Abbreviations: 6MWD, 6-min walk distance; BNP, B-type natriuretic peptide; CI, cardiac index; CO, cardiac output; mPAP, mean pulmonary arterial pressure; PAWP, pulmonary arterial wedge pressure; PVR, pulmonary vascular resistance; RAP, right atrial pressure; WHO-FC, World Health Organization functional class.

Dose distribution patterns are summarized in Supplementary Table 1. In the selexipag group, the most frequently administered dose was 3.2 mg/day (*n* = 7), and 46.7% of patients reached the maximum recommended dose, with a mean daily dose of 2.17 mg/day. In the riociguat group, the most common dose was 7.5 mg/day (*n* = 8), and 53.3% of patients reached the maximum dose, with a mean daily dose of 5.7 mg/day.

Both treatments were generally well tolerated, and no treatment discontinuations or serious adverse events were observed. In the selexipag group, adverse effects were reported in 9 patients (60%), most commonly gastrointestinal symptoms such as diarrhea (*n* = 6, 40%), followed by headache (*n* = 2, 13%) and hypotension (*n* = 1, 7%). Dose reduction was required in 2 patients (13%). In the riociguat group, adverse effects occurred in 7 patients (47%), including hypotension (*n* = 3, 20%), headache (n = 2, 13%), dizziness (n = 1, 7%), and peripheral edema (n = 1, 7%); dose reduction was required in 1 patient (7%).

### 3.2 Hemodynamic changes with selexipag

Selexipag treatment resulted in significant improvements in pulmonary hemodynamics. mPAP decreased from 39.0 ± 7.7 to 33.6 ± 8.7 mmHg (*P* = 0.008) and PVR decreased from 8.07 ± 3.4 to 6.69 ± 3.2 Wood units (WU) (*P* = 0.001). In contrast, CO and CI remained unchanged (CO: 4.19 ± 1.2 to 4.22 ± 1.0 L/min, *P* = 0.91; CI: 2.50 ± 0.6 to 2.51 ± 0.5 L/min/m^2^, *P* = 0.94), indicating that the hemodynamic benefits were driven mainly by reductions in mPAP and PVR, as shown in Table 2 and Supplementary Fig. 1.

**Table 2:** Hemodynamic parameters before and after Selexipag vs. Riociguat treatment.

Parameter	Selexipag Pre	Selexipag Post	Change	P-value	Riociguat Pre	Riociguat Post	Change	P-value
PAP (mmHg)								
Mean	**39.0 ± 7.7**	**33.6 ± 8.7**	**−5.4 ± 6.7**	**0.008**	**40.8 ± 9.3**	**33.0 ± 7.7**	**−7.8 ± 8.5**	**0.003**
Systolic	**62.1 ± 15.0**	**50.7 ± 15.2**	**−11.4 ± 11.8**	**0.002**	**67.1 ± 13.3**	**53.9 ± 15.0**	**−13.3 ± 16.2**	**0.007**
Diastolic	26.0 ± 4.6	23.5 ± 7.2	−2.5 ± 5.9	0.12	**25.7 ± 8.3**	**19.8 ± 5.0**	**−5.9 ± 5.8**	**0.002**
RAP (mmHg)	6.1 ± 2.0	5.3 ± 2.6	−0.8 ± 1.9	0.12	6.3 ± 3.7	5.3 ± 2.6	−0.9 ± 2.9	0.23
PAWP (mmHg)	8.4 ± 2.8	7.4 ± 2.7	−1.0 ± 2.6	0.16	8.7 ± 3.4	8.4 ± 2.6	−0.3 ± 3.2	0.75
CO (L/min)	4.19 ± 1.2	4.22 ± 1.0	+0.03 ± 1.0	0.91	**4.03 ± 0.8**	**4.84 ± 1.5**	**+0.81 ± 1.0**	**0.006**
SV (mL/beat)	59.8 ± 18.4	58.0 ± 19.2	−1.8 ± 11.6	0.55	**60.1 ± 14.9**	**70.8 ± 21.9**	**+10.7 ± 10.8**	**0.002**
HR (beats/min)	71.5 ± 10.1	75.5 ± 13.1	+3.9 ± 9.2	0.12	68.3 ± 9.0	69.3 ± 11.0	+1.1 ± 5.6	0.47
CI (L/min/m^2^)	2.50 ± 0.6	2.51 ± 0.5	+0.03 ± 0.6	0.94	**2.47 ± 0.4**	**2.90 ± 0.7**	**+0.42 ± 0.5**	**0.007**
SvO₂ (%)	60.8 ± 7.6	62.6 ± 6.5	+1.8 ± 7.7	0.39	61.5 ± 4.1	63.9 ± 5.3	+2.4 ± 5.1	0.09
SaO₂ (%)	90.6 ± 5.8	90.1 ± 5.8	−0.6 ± 5.3	0.68	89.7 ± 4.3	89.0 ± 3.9	−0.7 ± 3.4	0.45
PVR (WU)	**8.07 ± 3.4**	**6.69 ± 3.2**	**−1.38 ± 1.2**	**0.001**	**8.18 ± 3.0**	**5.61 ± 2.8**	**−2.57 ± 2.4**	**0.001**
TPR (WU)	**9.77 ± 3.4**	**8.06 ± 2.7**	**−1.71 ± 1.6**	**0.003**	**10.94 ± 4.2**	**7.41 ± 2.8**	**−3.53 ± 3.5**	**0.002**

Values are presented as mean ± standard deviation. Values in bold indicate significant differences (*P* < 0.05). Abbreviations: CI, cardiac index; CO, cardiac output; HR, heart rate; PAWP, pulmonary arterial wedge pressure; PAP, pulmonary arterial pressure; PVR, pulmonary vascular resistance; RAP, right atrial pressure; SaO₂, arterial oxygen saturation; SV, stroke volume; SvO₂, mixed venous oxygen saturation; TPR, total pulmonary resistance.

### 3.3 Hemodynamic changes with riociguat

Riociguat also significantly improved pulmonary hemodynamics. mPAP decreased from 40.8 ± 9.3 to 33.0 ± 7.7 mmHg (*P* = 0.003), and PVR decreased from 8.18 ± 3.0 to 5.61 ± 2.8 WU (*P* = 0.001). Notably, riociguat increased CO and CI (CO: 4.03 ± 0.8 to 4.84 ± 1.5 L/min, *P* = 0.006; CI: 2.47 ± 0.4 to 2.90 ± 0.7 L/min/m^2^, *P* = 0.007), which were driven predominantly by an increase in SV, with HR remaining unchanged, as shown in Table 2 and Supplementary Fig. 1.

### 3.4 Comparison between selexipag and riociguat

Between-group comparisons of Δ-values showed that reductions in mPAP and PVR were not significantly different between the selexipag and riociguat groups (mPAP: −5.4 ± 6.7 vs. −7.8 ± 8.5 mmHg, *P* = 0.40; PVR: −1.38 ± 1.2 vs. −2.57 ± 2.4 WU, *P* = 0.11) based on independent *t*-tests (Table 3). In contrast, riociguat produced significantly greater improvements in forward-flow parameters. CO increased by +0.81 ± 1.0 L/min with riociguat versus +0.03 ± 1.0 L/min with selexipag (*P* = 0.04). Consistent with this, SV and CI also improved significantly more in the riociguat group (SV: +10.7 ± 10.8 vs. −1.8 ± 11.6 mL, *P* = 0.005; CI: +0.42 ± 0.5 vs. +0.03 ± 0.6 L/min/m^2^, *P* = 0.048) (Table 3).

**Table 3:** Comparison of hemodynamic changes from baseline to post-treatment between selexipag and riociguat groups.

		**Selexipag**		**Riociguat**		**P-value**
		Change		Change		
PAP (mmHg)						
Mean		−5.4 ± 6.7		−7.8 ± 8.5		0.40
Systolic		−11.4 ± 11.8		−13.3 ± 16.2		0.72
Diastolic		−2.5 ± 5.9		−5.9 ± 5.8		0.13
RAP (mmHg)		−0.8 ± 1.9		−0.9 ± 2.9		0.88
PAWP (mmHg)		−1.0 ± 2.6		−0.3 ± 3.2		0.50
CO (L/min)		**+0.03 ± 1.0**		**+0.81 ± 1.0**		**0.040**
SV (mL/beat)		**−1.8 ± 11.6**		**+10.7 ± 10.8**		**0.005**
HR (beats/min)		+3.9 ± 9.2		+1.1 ± 5.6		0.31
CI (L/min/m^2^)		**+0.03 ± 0.6**		**+0.42 ± 0.5**		**0.048**
SvO₂ (%)		+1.8 ± 7.7		+2.4 ± 5.1		0.80
SaO₂ (%)		−0.6 ± 5.3		−0.7 ± 3.4		0.95
PVR (WU)		−1.38 ± 1.2		−2.57 ± 2.4		0.11
TPR (WU)		−1.71 ± 1.6		−3.53 ± 3.5		0.09

Values are presented as mean ± standard deviation. Values in bold indicate significant differences (P < 0.05). Abbreviations: CI, cardiac index; CO, cardiac output; HR, heart rate; PAWP, pulmonary arterial wedge pressure; PAP, pulmonary arterial pressure; PVR, pulmonary vascular resistance; RAP, right atrial pressure; SaO₂, arterial oxygen saturation; SV, stroke volume; SvO₂, mixed venous oxygen saturation; TPR, total pulmonary resistance.

To ensure the robustness of the between-group comparisons, ANCOVA models adjusting for the baseline values of each hemodynamic variable and key clinical covariates (age, sex, WHO-FC, and the interval between hemodynamic assessments) were additionally performed. These adjusted analyses confirmed the absence of significant differences in mPAP and PVR improvement while preserving the significant superiority of riociguat in the improvement of CO, CI, and SV (Table 4). No significant between-group differences were observed in RAP, PAWP, SvO₂, or SaO₂.

**Table 4:** Adjusted hemodynamic changes between groups (ANCOVA results).

	**Selexipag** **Adjusted Δ (Mean ± SE)**	**Riociguat** **Adjusted Δ (Mean ± SE)**	**Difference** **(Riociguat − Selexipag)**	**95% CI for Difference**	**P-value**
PAP (mmHg)					
Mean	−5.25 ± 1.95	−7.95 ± 1.95	−2.71	−8.67 to +3.26	0.36
Systolic	−11.70 ± 3.78	−12.96 ± 3.78	−1.26	−12.86 to +10.33	0.82
Diastolic	−2.26 ± 1.40	−6.14 ± 1.40	−3.88	−8.11 to +0.36	0.07
RAP (mmHg)	−1.06 ± 0.49	−0.68 ± 0.47	+0.37	−1.08 to +1.83	0.60
PAWP (mmHg)	−1.10 ± 0.65	−0.16 ± 0.65	+0.94	−1.02 to +2.90	0.33
CO (L/min)	**−0.10 ± 0.25**	**+0.95 ± 0.25**	**+1.05**	**+0.30 to + 1.79**	**0.008**
SV (mL/beat)	**−3.55 ± 2.69**	**+12.43 ± 2.69**	**+15.98**	**+7.84 to + 24.11**	**<0.001**
HR (beats/min)	+3.98 ± 2.20	+1.02 ± 2.20	−2.96	−9.68 to +3.75	0.37
CI (L/min/m^2^)	**−0.05 ± 0.13**	+**0.49 ± 0.13**	**+0.54**	**+0.14 to + 0.94**	**0.011**
SvO₂ (%)	+1.32 ± 1.36	+2.86 ± 1.36	+1.54	−2.59 to +5.67	0.45
SaO₂ (%)	−0.06 ± 1.01	−1.22 ± 1.01	−1.16	−4.20 to +1.89	0.44
PVR (WU)	−1.28 ± 0.51	−2.67 ± 0.51	−1.39	−2.92 to +0.14	0.07
TPR (WU)	−1.94 ± 0.62	−3.34 ± 0.55	−1.40	−3.20 to +0.39	0.12

Bold indicates statistical significance (P < 0.05). Difference (Riociguat − Selexipag) represents adjusted group effect. ANCOVA models were adjusted for age, sex, WHO functional class, time interval between baseline and pre-BPA right heart catheterization, and baseline value of each hemodynamic parameter. Residuals satisfied normality assumptions (Shapiro–Wilk *P* > 0.05 for all models). Abbreviations: BPA, balloon pulmonary angioplasty; CI, cardiac index; CO, cardiac output; HR, heart rate; PAWP, pulmonary arterial wedge pressure; PAP, pulmonary arterial pressure; PVR, pulmonary vascular resistance; RAP, right atrial pressure; SaO₂, arterial oxygen saturation; SvO₂, mixed venous oxygen saturation; SV, stroke volume; TPR, total pulmonary resistance.

## 4 Discussion

### 4.1 Hemodynamic effects of selexipag and comparison with riociguat

This retrospective study provides exploratory, hypothesis-generating evidence on the detailed hemodynamic effects of selexipag monotherapy in newly diagnosed, treatment-naïve patients with inoperable CTEPH prior to their initial BPA session. In this homogeneous cohort without prior PEA/BPA or other PH-targeted therapies, selexipag significantly reduced mPAP and PVR without major adverse effects, and approximately half of the patients were successfully titrated to the maximum recommended dose.

Both agents reduced mPAP and PVR, with no statistically significant between-group differences in this exploratory cohort. However, riociguat resulted in a significantly greater increase in CO, driven primarily by improved SV, whereas HR remained unchanged. This difference aligns with pharmacological distinctions between the prostacyclin receptor agonist pathway and soluble guanylate cyclase stimulation [15], [22], [23], [24]. From a clinical standpoint, this suggests that riociguat may be more suitable for patients with low-output states or right ventricular compromise, while selexipag may be considered when vasodilation is desired with preservation of systemic hemodynamics.

### 4.2 Influence of prior interventions and treatment context

Prior studies in inoperable CTEPH commonly included patients who had already undergone BPA or PEA and were receiving concomitant pulmonary vasodilators, limiting the ability to isolate drug-specific hemodynamic effects [17], [18], [25]. To our knowledge, the present study is the first to exclusively evaluate selexipag monotherapy in treatment-naïve patients. The favorable mPAP and PVR responses observed here contrast with the global SELECT trial, which was terminated for futility in a heterogeneous population with frequent prior BPA/PEA and background pulmonary vasodilators [25]. Similarly, in the Japanese Phase 3 trial [18], the majority of patients had undergone BPA and/or PEA or had received other pulmonary vasodilators, and the hemodynamic improvements were more prominent in CO than in mPAP reduction (Supplementary Table 2).

Baseline demographics and hemodynamics were generally comparable between our cohort and the Japanese Phase 3 population [18], including age (66.5 ± 10.6 vs. 66.3 ± 11.1 years), female proportion (66.7% vs. 74.4%), mPAP (both ∼39 mmHg), and CI (2.50 ± 0.6 vs. 2.44 ± 0.60 L/min/m^2^). These similarities indicate that initial disease severity and right ventricular function were largely comparable, suggesting that the discrepant hemodynamic response patterns are unlikely to reflect differences in the pharmacological effects of selexipag. Instead, marked differences in treatment context—particularly the absence of prior BPA/PEA or background endothelin receptor antagonist, phosphodiesterase type 5 inhibitor, or riociguat therapy in our cohort, versus 52.6% BPA, 12.8% PEA, and 66.7% background vasodilator therapy in the Japanese Phase 3 study—likely contributed to the predominant mPAP/PVR reduction seen in treatment-naïve patients, contrasted with the CO-dominant improvement in post-interventional cohorts.

### 4.3 Heterogeneity of treatment response across populations and phenotypes

Discrepancies in selexipag's reported efficacy across studies may also be partly attributed to heterogeneity in clinical background and disease phenotype. CTEPH-specific evidence suggests that hemodynamic responses differ according to prior PEA/BPA and background pulmonary vasodilator therapy, as demonstrated in the Japanese Phase 3 trial [18], while the SELECT randomized trial indicates that the timing and frequency of BPA may modify treatment effects in CTEPH [25].

Moreover, PAH class-effect data from the GRIPHON study indicates that diagnostic timing and clinical phenotype influence the treatment benefit of selexipag [16]. In addition, post hoc analyses suggest that comorbidity burden may further modify prostacyclin-pathway responsiveness [26]. These results support our interpretation that the heterogeneity of hemodynamic response in CTEPH patients is likely driven by background characteristics rather than inconsistencies in selexipag pharmacodynamics.

In addition, epidemiological and pathophysiological differences between Japan and Western countries should be considered. Compared with Western CTEPH cohorts, Japanese CTEPH cohorts have a higher proportion of women, fewer cases with documented deep vein thrombosis or acute pulmonary embolism, and a predominance of distal thromboembolic lesions [6], [8], [27], [28], whereas approximately 75% of patients in the international CTEPH registry have a previous history of acute pulmonary embolism [29]. These epidemiological differences may also influence treatment response and should be considered when generalizing our results.

Taken together, the directionality of mPAP and CO responses across studies likely reflects differences in prior interventions, background vasodilator therapy, disease stage, and underlying phenotype, rather than fundamental pharmacodynamic inconsistency. Accordingly, our findings should be interpreted as exploratory and hypothesis-generating but provide complementary insights into the potential role of selexipag as an initial therapy before BPA.

### 4.4 Clinical implications for pre-BPA hemodynamic optimization

The magnitude of hemodynamic improvement in the riociguat group was consistent with previous randomized trials such as MR-BPA and RACE [10], [11], further supporting its role as the first-line therapeutic agent in inoperable CTEPH. Of note, the RACE follow-up study demonstrated that pre-BPA riociguat therapy reduced BPA-related complications, potentially due to the reduction in mPAP before intervention [11]. Because selexipag was associated with reductions in mPAP in this population, it may theoretically offer procedural benefits, although this hypothesis requires validation in larger prospective randomized trials.

Clinical implications differ between agents. Riociguat may be favored in patients requiring rapid improvement in CO or those with severely compromised right ventricular function, although its use may be limited in patients with hypotension or nitrate therapy due to the risk of systemic vasodilation [22], [23]. Conversely, selexipag may be considered, particularly in patients with contraindications to, or intolerance of, riociguat. Although the selexipag group demonstrated significant reductions in mPAP and PVR, CO did not improve, and we did not assess functional outcomes, right ventricular function, and BPA-related outcomes. Therefore, selexipag may best be interpreted as providing supportive pre-BPA optimization aimed at unloading the right ventricle, rather than affording a stand-alone prognostic benefit.

The concurrent use of selexipag and riociguat has been conceptually proposed; however, evidence remains limited, and caution is needed due to the potential for additive systemic vasodilation. Further research is warranted to determine optimal sequencing strategies and the feasibility of combination therapy.

The safety and tolerability profiles of selexipag were consistent with earlier CTEPH and PAH studies [16], [17], [18]. Approximately half of the patients achieved the maximum recommended dose, and all other patients achieved the highest tolerated dose without discontinuation due to adverse effects, as detailed in Supplementary Table 1. Common adverse effects, such as diarrhea and headache, were manageable and did not require hospitalization or therapy withdrawal. Selexipag is administered orally using a standardized titration schedule defined in the prescribing information, and in our cohort dose escalation was generally feasible; however, adherence and logistical aspects were not evaluated and represent areas for future research.

### 4.5 Limitations

This study has several limitations. First, it was a single-center retrospective study with a small sample size, particularly in the selexipag group, and without randomized treatment allocation, which inherently limits causal inference and may introduce selection and indication bias. Dosing variability also existed across individuals, and differences in CO response may partly reflect titration tolerance rather than pharmacodynamic efficacy. In addition, no formal sample size calculation was performed, and the study may have been underpowered to detect clinically meaningful between-group differences in hemodynamic outcomes.

Second, the study population consisted exclusively of Japanese patients treated at a specialized center with extensive BPA expertise, which may limit the generalizability to other clinical environments or populations with different CTEPH phenotypes. Because patient demographics and disease phenotype in Japan may differ from those in Western countries, caution is needed when generalizing our findings to broader international populations.

Third, the follow-up duration was short and did not include assessments of functional capacity, right ventricular function, quality of life, or BPA-related outcomes. Thus, the long-term clinical relevance and safety profile of pre-BPA selexipag therapy remain unclear.

Given these limitations, including the small sample size, absence of randomization, and short-term observational design, these results should be interpreted cautiously. In addition, reductions in mPAP and PVR should be regarded as surrogate hemodynamic endpoints, and whether these changes translate into improved BPA-related efficacy, procedural safety, functional capacity, or long-term clinical outcomes remains uncertain. Larger prospective studies are required to confirm the clinical relevance of these hemodynamic changes and to clarify the optimal role of selexipag within the treatment pathway for inoperable CTEPH before BPA.

## 5 Conclusions

This exploratory retrospective study suggests that selexipag monotherapy is associated with reductions in mPAP and PVR in treatment-naïve patients with inoperable CTEPH prior to BPA, although improvements in cardiac output were less pronounced than with riociguat. Selexipag may represent a feasible option for pre-BPA hemodynamic optimization in selected patients, particularly when riociguat is contraindicated or not tolerated.

## Author contribution

NK was responsible for conceptualization, methodology, formal analysis, investigation, resources, data curation, original draft writing, review and editing, supervision, project administration, and funding acquisition.

TS and TSz contributed to investigation, resources, and review and editing of the manuscript.

FO, MA, UY, KS, SK, and SM contributed to resources and review and editing.

JH, MK, FOt, NI, TI, SKa, and KO contributed to manuscript review and editing.

MM and DU provided supervision and manuscript review and editing.

KH contributed to supervision, funding acquisition, and manuscript review and editing.

## CRediT authorship contribution statement

**Naohiro Komura:** Writing – review & editing, Writing – original draft, Supervision, Resources, Project administration, Methodology, Investigation, Funding acquisition, Formal analysis, Data curation, Conceptualization. **Teruyasu Sugano:** Writing – review & editing, Supervision, Resources, Investigation. **Toru Suzuki:** Writing – review & editing, Resources, Investigation. **Fumiaki Ono:** Writing – review & editing, Resources. **Maria Abe:** Writing – review & editing, Resources. **Uno Yamamoto:** Writing – review & editing, Resources. **Keisuke Sekiya:** Writing – review & editing, Resources. **Sho Kodama:** Writing – review & editing, Resources. **Shintaro Minegishi:** Writing – review & editing, Resources. **Shingo Kato:** Writing – review & editing. **Kozo Okada:** Writing – review & editing. **Junya Hosoda:** Writing – review & editing. **Masaaki Konishi:** Writing – review & editing. **Fumiyuki Otsuka:** Writing – review & editing. **Noriaki Iwahashi:** Writing – review & editing, Resources. **Tomoaki Ishigami:** Writing – review & editing. **Daisuke Utsunomiya:** Writing – review & editing, Supervision. **Makoto Mo:** Writing – review & editing, Supervision. **Kiyoshi Hibi:** Writing – review & editing, Supervision, Funding acquisition.

## Ethics approval

This study was approved by the Ethics Committee of Yokohama City University Hospital (approval number: F230500041; June 6, 2023) and adhered to the ethical guidelines of the 1975 Declaration of Helsinki. Research information and consent forms were provided to patients in an opt-out format after the investigation because of the retrospective nature of this study.

## Funding

This research did not receive any specific grant from funding agencies in the public, commercial, or not-for-profit sectors.

## Declaration of competing interest

The authors declare that they have no known competing financial interests or personal relationships that could have appeared to influence the work reported in this paper.

## Data Availability

The deidentified participant data will not be shared.

## Glossary

BPA: balloon pulmonary angioplasty
CI: cardiac index
CO: cardiac output
CTEPH: chronic thromboembolic pulmonary hypertension
HR: heart rate
mPAP: mean pulmonary arterial pressure
PAH: pulmonary arterial hypertension
PAWP: pulmonary arterial wedge pressure
PEA: pulmonary endarterectomy
PH: pulmonary hypertension
PVR: pulmonary vascular resistance
RAP: right atrial pressure
RHC: right heart catheterization
SaO₂: arterial oxygen saturation
SV: stroke volume
SvO₂: mixed venous oxygen saturation
WHO-FC: World Health Organization functional class
WU: Wood units

## Appendix A Supplementary data

Supplementary material Hemodynamic changes before and after treatment with selexipag or riociguat, dose distributions of each drug, and differences between the present study and major selexipag studies in patients with CTEPH.
