# Supplementary material for: Retrospective evaluation of Selexipag monotherapy on pulmonary hemodynamics in newly diagnosed, treatment-naïve patients with chronic thromboembolic pulmonary hypertension prior to balloon pulmonary angioplasty
Source: Int J Cardiol Heart Vasc. 2026 Jun 15;65:101956. doi: 10.1016/j.ijcha.2026.101956 (PMC13292260; doi:10.1016/j.ijcha.2026.101956)
Supplement: Supplementary file 1 — Supplementary material Hemodynamic changes before and after treatment with selexipag or riociguat, dose distributions of each drug, and differences between the present study and major selexipag studies in patients with CTEPH. [file mmc1.docx]

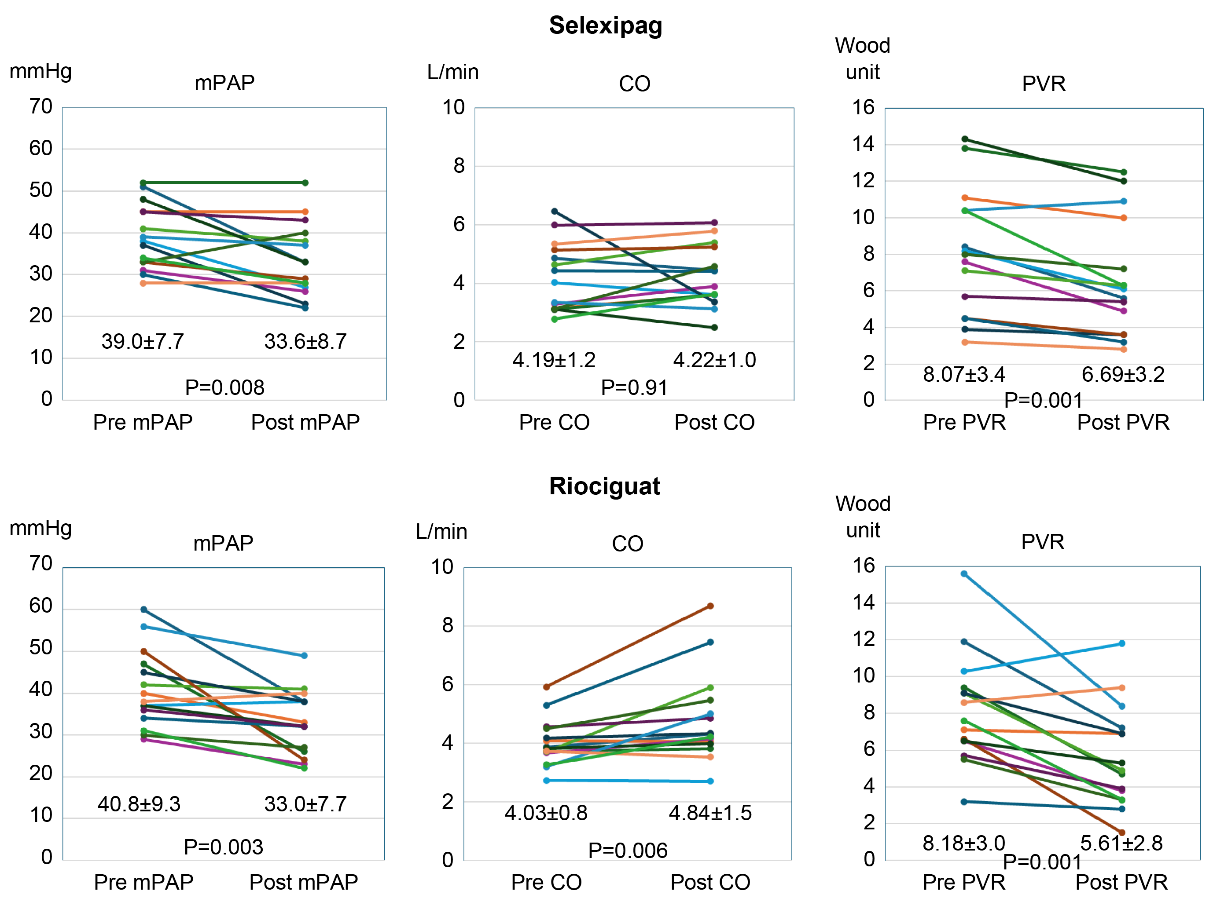
**Supplementary Figure 1.** Hemodynamic changes before and after treatment with selexipag and riociguat (n=15 per group). Abbreviations: CO, cardiac output; mPAP, mean pulmonary arterial pressure; PVR, pulmonary vascular resistance.

**Supplementary Table 1.** Dose distribution of each drug

| Selexipag (n=15) | | Riociguat (n=15) | |
| --- | --- | --- | --- |
| Dosage (mg/day) | n | Dosage (mg/day) | n |
| 0.8 | 4 | 1.5 | 1 |
| 1.2 | 2 | 3.0 | 3 |
| 2.2 | 1 | 4.5 | 2 |
| 2.4 | 1 | 6.0 | 1 |
| 3.2 | 7 | 7.5 | 8 |

**Supplementary Table 2.** Differences between the present study and major selexipag studies of CTEPH

|  | Study design | CTEPH after BPA | CTEPH after PEA | Other PAH drugs |
| --- | --- | --- | --- | --- |
| Present Study | Retrospective  (n=30) | 0% | 0% | 0% |
| Japanese Phase III  Ogo et al. Eur Respir J 2022;60:2101694 | RCT  (n=78) | 52.6% | 12.8% | 66.7% |
| Global Phase III  (SELECT Trial)  Kim et al. Eur Respir J 2024;64:2400193 | RCT  (n=91) | 22.0% | 47.3% | 74.5% |

Abbreviations: BPA, balloon pulmonary angioplasty; CTEPH, chronic thromboembolic pulmonary hypertension; PAH, pulmonary arterial hypertension; PEA, pulmonary endarterectomy; RCT, randomized controlled trial.
